# Developing standards for virtual delivery of mental health services in Canadian primary care: A qualitative study and modified Delphi process

**DOI:** 10.1371/journal.pmen.0000071

**Published:** 2024-10-17

**Authors:** Abban Yusuf, Ginetta Salvalaggio, Osnat Melamed, Stephanie Montesanti, Helen Atherton, Lucie Langford, Saadia Sediqzadah, Tamara Do Amaral, Braden O’Neill

**Affiliations:** 1 MAP Centre for Urban Health Solutions, Li Ka Shing Knowledge Institute, St. Michael’s Hospital, Toronto, Ontario, Canada; 2 Department of Family Medicine, University of Alberta, Edmonton, Alberta, Canada; 3 Department of Family and Community Medicine, Temerty Faculty of Medicine, University of Toronto, Toronto, Ontario, Canada; 4 Centre for Mental Health and Addiction (CAMH), Toronto, Ontario, Canada; 5 School of Public Health, University of Alberta, Edmonton, Alberta, Canada; 6 Primary Care Research, University of Southampton, Southampton, England; 7 Department of Psychiatry, St. Michael’s Hospital, MAP Centre for Urban Health Solutions, Li Ka Shing Knowledge Institute, Temerty Faculty of Medicine, University of Toronto, Toronto, Canada; 8 Population Health and Value-based Health Systems, Ontario Health, Toronto, Ontario, Canada; Instituto Federal do Maranhão: Instituto Federal de Educacao Ciencia e Tecnologia do Maranhão, BRAZIL

## Abstract

In Canada, public health measures necessitated by the COVID-19 pandemic resulted in a rapid onset and prolonged, widespread increase in the use of virtual primary care services, including for mental health conditions. Our aim was to develop standards on virtual delivery of mental health services in primary care in Canada using information obtained from an earlier rapid review as well as participant feedback obtained through interviews and a focus group. We developed standards using three interlinked processes. First, we completed a rapid review of guidelines regarding virtual primary mental health care services. We then invited health care workers and people with lived experience of mental health concerns to participate in a focus group and interviews. Finally, members of the study team drafted standards and shared them with an advisory group, who reviewed their feasibility, phrasing, and acceptability through a modified Delphi process. Standards ranked as having less than 100% feasibility and acceptability were brought to a virtual discussion of the advisory group to finalize the list. Seven participants were recruited into the focus group and interviews. We identified three themes: (i) patients’ and providers’ agreement about expectations regarding virtual care, (ii) accessibility and equity, and (iii) safety planning in the delivery of virtual care. We drafted 18 standards on virtual primary mental health care delivery that were reviewed by an advisory group of identified experts. Thirteen standards were included in the final list. The standards bring attention to continuity of care, and resources and information that should be given to patients to further health equity. These standards provide guidance for the organization and delivery of virtual mental health services in Canadian and international primary care, particularly within the context of single payer health systems.

## Introduction

### Background

As a result of the COVID-19 pandemic and related public health restrictions, virtual delivery of health care became far more common in high income nations and has persisted since [1]. Virtual care is "any interaction between patients and/or members of their circle of care, occurring remotely, using any forms of communication or information technologies…" [2]. In the first months of the COVID-19 pandemic in Canada, the usage of virtual care increased substantially; for example, there was a 5,600% increase in virtual visits in Ontario; virtual care made up more than 70% of primary care visits [3]. Between 2019 and 2020, only 4% of all physician-provided mental health services were delivered virtually; one year later, this percentage increased to 57% of all physician-provided mental health services [4, 5].

Approximately 20% of people in Canada will experience a mental health issue each year [6]. Mental health services in Canada are often first accessed through primary care [7], with the most common reasons for primary care visits being anxiety and depression [8, 9]. Yet, approximately 17% of Canadians–at least 6 million people–do not have access to primary care providers [10]. Given that virtual care services are proposed to improve access to primary health care for many people while decreasing the cost of delivering care when performed well [11, 12] and that psychiatry-based mental health care has been reported as particularly amenable to virtual care delivery compared to other forms of physical ailments [13], it is evident that virtual delivery of mental health services will be an important ongoing pillar of primary health care delivery. Thus, improving access to virtual care will be an important component of improving health equity within the Canadian health care system [12].

Substantial increases in virtual care use have resulted in an abundance of literature on virtual care service delivery [12–32]. Our team completed a rapid review of guidance and standards for virtual delivery of mental health services from high-income country settings and identified 40 resources from 9 countries [33]. Themes from content analysis of those resources included: screen patients for appropriateness of virtual care; obtain emergency contact details; communicate transparently with patients; improve marginalised patients’ access to care; support health equity for all patients; determine the cost-effectiveness of virtual care; inform patients of insurance coverage for virtual care services; increase provider training for virtual care and set professional boundaries between providers and patients [33]. Despite these resources being available in other countries, efforts to organise virtual care guidance and recommendations have been limited in Canada until recently [33]. Existing frameworks and guidelines for virtual care in Canada, such as the Canadian Medical Association’s 2021 ‘Virtual Care Playbook’ [34] and the Foundation of Medical Regulatory Associations of Canada’s 2021 guidance [35] are largely focused on operational considerations such as what size screen to use, or noting that the limitations of virtual care must be disclosed to patients. Existing guidance is therefore inadequate to support primary care providers to be able to provide safe, effective virtual care in general; and this guidance is not focused on mental health in particular, which has its own set of challenges including patient safety considerations that need to be specifically addressed. Various publications in the literature have already noted how poorly planned or delivered virtual health care appointments can increase the possibility of patients experiencing negative consequences. For example, one article found that patients who report to ‘walk-in’ virtual health visits (that is, episodic care settings where there is no expectation of longitudinal follow up) were more likely to visit an emergency room later [36]. Another article noted that the privacy of patient health information may be compromised if adequate protections are not put in place [28]. Some authors caution that virtual health care could worsen health inequities and disparities [12]; for example, in the United States, virtual health care services tend to be used more by people of higher income, while people of lower income use in-person visits more frequently [5].

Thus, it is necessary to develop standards for the delivery of virtual mental health services. Standards provide a benchmark for high quality care, and they provide guidance for practitioners and organizations enabling comparison with each other. According to Health Quality Ontario, “Quality standards outline for clinicians and patients what quality care looks like” [37]. Implementing standards can also ensure that quality care is delivered in a consistent manner for all patients by minimising variation [38].

Many research publications and provincial documents were quickly developed over the course of the COVID-19 pandemic to guide and support health care providers as they sought to adjust to the rapidly-changing health care landscape. Yet, despite the quickly growing popularity and usage of virtual care, there is a paucity of recommendations that emphasize mental health services in primary care, health equity and improvement of quality of care within a Canadian context. The objectives of this study were to develop and refine a list of standards on virtual delivery of mental health services in primary care in Canada using information from an earlier rapid review [33] as well as the participant feedback obtained in this study through interviews and a focus group.

## Methods

### Study design

**Fig 1:**
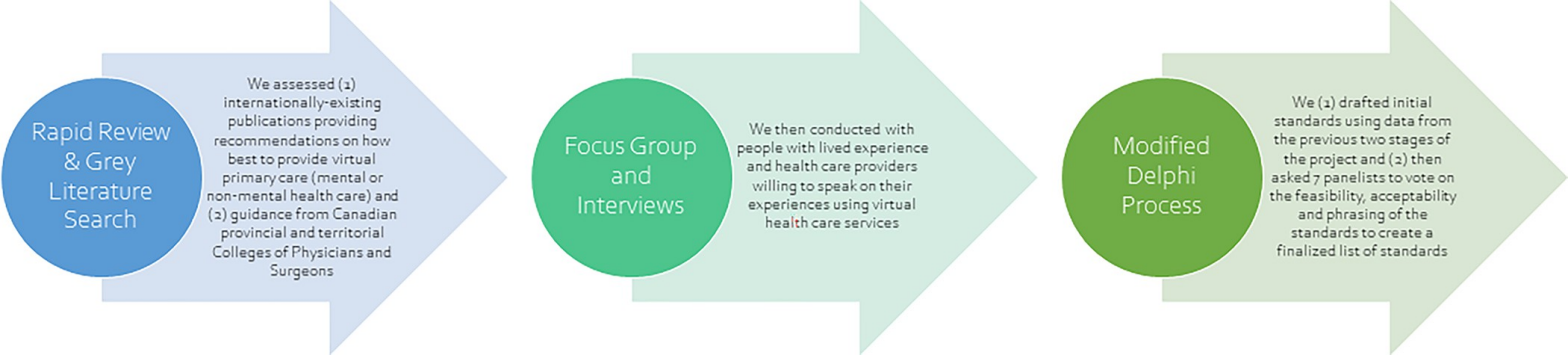
Project overview.

We conducted a qualitative study using a modified Delphi process which included findings from a rapid review [33], consultation with people with lived experience and health workers, expert review from an external advisory group, and development of consensus among the study team. (Fig 1) The study was mostly conducted virtually; all meetings were conducted over Zoom. This manuscript has been reported using the Consensus Reporting Items for Studies in Primary Care (CRISP) checklist [39], located in S1 Table.

#### Ethics statement

This project was approved by the Unity Health Toronto Research Ethics Committee (approval #22–262). Informed consent was obtained verbally prior to the focus group and interviews.

#### Focus group and interviews

We used the findings of an earlier rapid review of guidance from high-income country settings [33] to develop questions for a focus group and a series of interviews with healthcare providers and people with lived experience. (S1 Text). We recruited seven participants into one focus group and two interviews (assignment based on a participant’s request; they could choose to be questioned individually in an interview or as part of a group).These lasted about one hour each, and were held between May and July, 2023 on Zoom; we used the internal features of Zoom to record each interview and to generate draft transcripts, which a research staff member reviewed and edited in detail for accuracy for use in analysis. Data were stored on a secure server at Unity Health Toronto.

#### Analysis of focus group and interview data

Three team members (BO, NE, AY) used thematic analysis to code the focus group and interview transcripts [40]. This involved three phases: (1) reading of the data, (2) coding of the data, which allowed for early data management and analysis, and (3) theming, where the team members shared their perspectives, deliberated and grouped codes into themes (42). In the first phase, reading, all team members familiarised themselves with the transcripts by reading through them individually. In the second phase, coding, the team members created “codes” for each transcript, defined by Saunders et al. as “labels for concepts in the data that are directly relevant in the study objective”, and recorded these codes and their definitions. After individual coding was completed for the first transcript in Microsoft Word, BO, NE and AY met to negotiate consensus and develop these codes into a shared codebook to use for the two subsequent transcripts [13]. Lastly, in the third phase, theming, the three team members individually grouped together repeating codes and constructs into overarching themes, using Microsoft Excel. They then met together to deliberate and decide on a final set [40].

Sample quotes were identified to exemplify key themes during this phase. Following a virtual discussion, all members of the study team had the opportunity to review the themes and offer feedback on the data interpretation. Data analysis was completed using Microsoft Word and Excel.

#### Delphi consensus process

Three study team members (AY, NE, BO) drafted an initial list of standards (containing 18 standards in total) using information from the rapid review (specifically the themes described in the manuscript) [33] and the results of the analysis of the focus group and interviews. We then conducted a modified Delphi consensus process by sending these to the 14 members of the advisory group [41].

In a typical Delphi process, a group of expert panel members is asked to repeatedly, independently, and anonymously rank and evaluate statements on a given topic [42, 43]. In subsequent rounds, the anonymized responses from the previous rounds are shared with panelists, to give panelists the opportunity to read other opinions and decide whether to alter their own (43). The format of the Delphi process is designed to attain a group’s consensus on a set of drafted statements while minimising bias that may originate from group conformity [42].

In this study, we implemented a modified two-step Delphi consensus process, drawing inspiration from the method used by Eubank et al [41]. Using this methodology, we first prepared a Google Forms-based survey asking for independent feedback on the agreeability, feasibility, and phrasing of the initial draft of standards. We sent this to the 14 members of our advisory group. Seven people from the advisory group responded; this group comprised researchers, physicians, health care providers and people with lived experience who did not participate in drafting the original standards. None of the authors who originally drafted the standards took part in this Delphi process.

We modified the Delphi approach by having the advisory group conduct the process to combine and condense the initial list, rather than sending out a survey more broadly for prioritization. We did this because of resource and time limitations within our one-year study period. Once feedback from this round was put together, the study team met in an online video meeting to go over the feedback and make final revisions to the list of proposed standards. Any standard that had a score of less than 100% for both feasibility and agreeability was brought to that meeting. Five members attended.

*Study team*. The study team was based at a research hospital based in Toronto, Ontario, Canada. The authors GS, OM, SM, HA, LL and BN were involved in the conception of the study and methodology design. The focus group and interviews were conducted by a research coordinator with experience in public health (NE) and the principal investigator (BO) who is a family physician and health services researcher. We were cognizant of power differentials particularly within interactions between us as research professionals and people with lived experience of mental illness. Although we sought to limit the effect of this on people’s responses by recruiting participants for the focus group and interviews with whom we did not have any pre-existing relationship, it remains possible that people may have been inhibited in sharing criticism of their own virtual care experiences.

*External advisory group*. We established an external advisory group comprising 14 primary care providers, health care and mental health services researchers and professionals and people with lived experience of mental illness. This group provided guidance over the course of the project and acted as external consultants to the authors.

### Participants

We invited individuals through our personal networks and connections from various backgrounds across Canada, including people with lived experience, health care providers, policy makers, administrators, and clinicians, to participate in interviews, focus group and the advisory group (who completed the Delphi process) to discuss their views on virtual primary mental health care services and review the standards. We also conducted a Google search to identify at least one mental health organisation in every province and territory and contacted these organisations (19 in total) through email to recruit participants with experience in delivering or receiving mental health care. Participants in the focus group and interviews were provided with a $50 Canadian gift card as reimbursement for their time and participation in the study.

## Results

### Focus group

Participants included: one psychotherapist, four people with lived experience of mental illness, one social worker and one psychiatrist. Two participants were from Alberta, two from British Columbia and three from Ontario.

Four people with lived experience and one health care provider met in the focus group. Each of the two interviews included one health care provider. All of the participants were adults (18 years or older) who self-identified as people with lived or living experience of mental illness, mental health clinicians, policy makers or health care administrators.

Codes assigned from analysis were developed into three themes: (1) patients and providers agreeing about expectations regarding virtual care, (2) accessibility and equity, and (3) safety planning in virtual care.

### Patients and providers agreeing about expectations regarding virtual care

Several topics were covered during the focus group and interviews, including modality preferences (e.g., videocall versus telephone call) and the usage of asynchronous care, primarily for communication and appointment planning. There was substantial diversity of opinion on the optimal modality for virtual care. Some participants reported preferring telephone to video, expressing that telephone calls aided them by allowing:

“…[them] to tune into the voice that much easier […without…] think[ing] about [their] own pair of verbals or body language…”. [Health care provider, Focus group 1]

Other participants expressed that maintaining communication through body language is a vital part of care, stating they:

“… read people’s body language, their tone of voice…” [Person with lived experience, Focus group 1],

during appointments. One participant noted that the modality used ultimately depends on the patient and their needs and preferences:

“But in the end, it’s also the person’s choice and there are a few people who after the initial assessment, don’t want to come back in [person] if they can avoid it.” [Health care provider, Interview 2].

Some providers expressed concerns about how much time they may be expected to invest into communicating asynchronously with their patients, noting that asynchronous care is better for quick communication but not for serious, complex conversations. One participant expressed:

“If it’s a question about something, we’ve got no problem like about a resource or something fair. But if I have to spend more than 10 minutes engaged in responding to or looking something up for you, then there will be a charge associated with that.” [Health care provider, Interview 1].

### Accessibility and equity

Although participants commented on the benefits of virtual care, they also discussed gaps in the accessibility and equity of these services. For instance, one participant described how virtual appointments could improve privacy in their own life:

“… I felt like there was less shame too, because I didn’t have to tell my workplace where I was going, I could sort of schedule it on my own time. And it felt more personalized to me doing it from my own home.” [Person with lived experience, Focus group 1].

Another participant pointed out that virtual care is still inaccessible for some:

“… a lot of people […] wouldn’t necessarily have access to data in their phones …” [Person with lived experience, Focus group 1].

Another participant brought attention to the fact that virtual primary care appointments are impossible to set up without first having access to a primary care physician. They wondered:

“…about the situation where people ‘don’t have a primary care physician, or they ‘don’t have a primary care clinic necessarily. So, they ‘don’t have access, in a sense …” [Health care provider, Focus group 1].

### Safety planning in virtual care

The final theme was safety planning in virtual care. Conversations on this theme described situations in which virtual care may be riskier to a patient compared to in-person appointments. For example, one participant suggested it may be easier on a patient to have certain conversations in-person rather than virtually, stating that they would advise their patients to meet in-person for follow-up if something traumatic came up during a video call:

“…, there are things that you discover, during the phone visit, that you may not be aware of at the beginning, or you may not have considered at the beginning…” [Health care provider, Interview 2].

Participants also discussed emergency situations, where a patient is experiencing a crisis at a different location from their provider. One participant described:

“…if someone’s at such a high crisis point, and they cannot be grounded, virtually, it puts you in a really kind of sticky spot, because although we may have their emergency contact information, you know, to call someone in the event of- I find not being able to kind of make that eye contact, not being able to see that they’re doing the exercise properly… anxiety provoking for the clinician as well.” [Health care provider, Focus group 1].

Participants also discussed potential strategies to minimise these risks. For example, participants believed it was important to develop and implement a plan for emergencies:

“…make the emergency plan explicit…have an explicit conversation about what’s going to happen if you’re at risk, and you’re in crisis, and what are we going to do about it?” [Health care provider, Focus group 1].

### Delphi consensus process and standards development

After the focus group, interviews and rapid review (34) were completed, three study team members (NE, AY, BO) developed a draft list of 18 standards. Seven advisory group members (who had not taken part in drafting the standards initially) took part in the modified Delphi consensus process to rate the agreeability and feasibility of the 18 draft standards. The number of participants between the first and second rounds of the Delphi process dropped from seven to five. These advisory group members self-identified as four researchers, two health care administrators, two healthcare providers and one person with lived experience. (People could choose more than one role.) The initial draft list of standards and the feedback provided on them are presented in Table 1.

**Table 1:** Study team ratings on agreeability and feasibility of initial draft standards.

Statement Number	Statement	Agreeability (n (%))	Feasibility (n (%))
**1**	All patients should have a standardized assessment of symptoms and severity completed prior to or during their appointment, if presenting with symptoms for which standardized assessments exist	7 (100%)	6 (85.7%)
**2**	All staff interacting with patients (including primary care providers, clerical staff, nurses, etc.) should receive specific training about how to conduct virtual care appointments for mental health issues, including: technical aspects of care, appropriateness, guidelines and ethics	7 (100%)	7 (100%)
**3**	All patients should receive a list of local crisis resources either during or after an appointment for mental health concerns	7 (100%)	6 (85.7%)
**4**	Anticipated response time (how long patients can expect to wait between communicating with their provider and receiving a response) should be agreed on between providers and patients and documented in the medical record	7 (100%)	6 (85.7%)
**5**	An emergency contact should be documented in the medical record of anyone accessing virtual mental health care, to be used in case the patient abruptly disconnects or discloses information that results in concern about imminent harm	7 (100%)	7 (100%)
**6**	Providers should ask patients during virtual mental health appointments whether they are in a setting that is sufficiently private for them to be comfortable continuing with the appointment	7 (100%)	7 (100%)
**7**	Patient screening for virtual mental health care should include asking patients about their preference for modality (in-person, phone, video)	7 (100%)	6 (85.7%)
**8**	All patients should be informed of safety protocols—for example, what will happen if they hang up in the middle of an appointment—in place prior to commencing virtual mental health care	7 (100%)	7 (100%)
**9**	Primary care providers and patients should discuss whether patients intend to record virtual mental health appointments, and the outcome of this conversation should be documented in the medical record	4 (57.1%)	5 (71.4%)
**10**	If disclosure by a patient of substantially traumatic events is anticipated, an in-person appointment should be recommended in the first instance instead of a virtual modality	5 (71.4%)	3 (42.9%)
**11**	Patients and providers should discuss whether limited visibility of body language (either through telephone or video calls) could impact the quality of assessment that can be provided, and should collaboratively establish a plan for future care if this is anticipated to be a substantial barrier	6 (85.7%)	5 (71.4%)
**12**	Patients without access to longitudinal primary care should still be able to access virtual mental health care and services through episodic care options such as ’virtual walk-in clinics’	5 (71.4%)	6 (85.7%)
**13**	Peer support opportunities should be offered to older patients who disclose during a virtual mental health consultation that they are experiencing psychological distress	5 (71.4%)	4 (57.1%)
**14**	American Sign Language—trained providers should be available for Deaf patients accessing virtual mental health care	7 (100%)	5 (71.4%)
**15**	Patients from equity-seeking groups should have opportunities for prioritized access to virtual mental health care services	5 (71.4%)	4 (57.1%)
**16**	Virtual mental health care services provided to patients with mental health concerns should be appropriate and cognizant of the needs and preferences of both patients and providers	6 (85.7%)	6 (85.7%)
**17**	Patients with mental health concerns should have access to clear, unambiguous information about what virtual mental health services are available and covered by medicare in their province/territory	7 (100%)	5 (71.4%)
**18**	Patients should be provided with resources to support safe and effective use of technology for virtual mental health care, such as guidance for what is an appropriate setting for an appointment	6 (85.7%)	4 (57.1%)

Out of the 18 draft standards, only four (22.2%) were unanimously rated both agreeable and feasible (Standards 2,5,6 and 8). 10 standards (55.6%) were unanimously rated agreeable (Standards 1–8, 14, 17), and four (22.2%) standards were unanimously rated feasible (Standards 2, 5, 6, 8).

The study team then made final revisions to the draft standards; these revisions included modifying phrasing of statements, deleting statements entirely and combining statements together. Five draft standards were left unmodified. Three draft standards were combined into one. Wording was revised for seven draft standards. Three draft standards were deleted. After these revisions were made, the standards were brought back to the study team, who made minor changes to their wording.

The final list of standards developed from this process are presented in Table 2.

**Table 2:** Finalized list of standards for virtual mental health care delivery in primary care.

Standard Number	Standards
**1**	All patients should have a standardized assessment of symptoms and severity completed as needed during or after every virtual primary care mental health appointment, if presenting with symptoms/conditions (such as anxiety or depression) for which standardized assessments exist
**2**	All primary care staff interacting with patients (including family physicians and other primary care providers, clerical staff, nurses, etc.) should receive specific training about how to conduct virtual care appointments for mental health concerns, including technical aspects of care, appropriateness, guidelines and ethics
**3**	Anticipated response time (how long patients can expect to wait between communicating with their provider and receiving a response) should be discussed between primary care providers and patients and the results of that discussion should be documented in the medical record
**4**	All patients accessing virtual primary care mental health services should be made aware of the location of a list of local crisis resources, an emergency contact should be documented in the medical record, and a discussion should occur about what will happen if there is a sudden disconnection or concerns related to imminent harm
**5**	Providers should ask patients during virtual primary care mental health appointments whether they are in a setting that is sufficiently private for them to be comfortable continuing with the appointment, or whether they would prefer to reschedule to another time or modality. They should document this in the medical record
**6**	Patient screening for virtual primary care mental health appointments should include asking patients about their preference for modality (e.g., in-person, phone, video) which a provider will try to accommodate, where possible
**7**	Primary care providers and patients should discuss whether patients intend to record virtual mental health appointments, and the outcome of this conversation should be documented in the medical record
**8**	If disclosure by a patient of traumatic events is anticipated, a benefit-risk discussion should be conducted and the patient should be given the option of an in-person appointment
**9**	While continuity of care in virtual primary care mental health services is important, if this is not available, episodic delivery may be required. In this instance, the episodic virtual care provider should recommend/determine a plan of care which could facilitate continuity of care being provided
**10**	Simultaneous translation (for synchronous care) and text translation (for asynchronous care) should be available for everyone, including where American Sign Language-trained providers are needed for Deaf patients accessing virtual primary care mental health services
**11**	Patients from equity-seeking groups should have opportunities for prioritized access to virtual mental health services, if that is their preference and it is feasible
**12**	Patients with mental health concerns should have access to clear, unambiguous information about what virtual mental health services are available and covered by their provincial/territory health insurance
**13**	Patients should be provided with resources to support safe and effective use of technology and digital literacy for virtual mental health care, such as guidance for what is an appropriate setting for an appointment

## Discussion

We developed a list of standards that can be used nationally and serve as a blueprint for delivering virtual mental health services in primary care. This process was comprehensive and drew on previous research, feedback from interviews and a focus group with community members and health care workers and input from a diverse group of panelists. Although this project was focused on Canadian primary care, we believe these standards are applicable to other single-payer healthcare settings.

Data from the interview and focus group led to the development of three themes: (1) patients and providers agreeing about expectations regarding virtual care, (2) accessibility and equity, and (3) safety planning in virtual care. These themes were used in the development of the drafted standards 4–12, 14, and 15; findings from the rapid review [33] were used to draft the other standards. There were some similarities between these standards and guidance that has been developed in other settings. For example, Ontario Health (which oversees the administration of the health system in Ontario, Canada’s most populous province) published a guidance document emphasizing the need for health equity, assessing population health needs and determining the best modalities of virtual care for patients [31]. Other literature exploring how to improve delivery of virtual mental health care in Canada also described the importance of considering the impact of health care disparities, including those experienced by populations experiencing inequitable access to quality care, during guideline development [44]. This is represented in standard 11 (“Patients from equity-seeking groups should have opportunities for prioritized access to virtual mental health services, if that is their preference and it is feasible”) (Table 2).

As part of this overall research project, we reviewed Canadian provincial and territorial policies on delivering virtual health care [45] and found some other similarities and differences to the standards we created. For example, standards of practice published online by the College of Physicians and Surgeons of Alberta stated those providing virtual care must assess the suitability of their patient’s physical environment, obtain consent, develop plans for adverse or emergency situations and confirm the location of their patient prior to initiating each virtual appointments [46], consistent with standards 4 and 5 in our study (Table 2). The College of Physicians and Surgeons of Saskatchewan provided similar guidance through their virtual care policy, and reported that physicians providing virtual care should, among other things, strive to ensure confidentiality and verify the appropriateness of virtual care with each patient before commencing a virtual appointment [47].

Some of the standards were developed from the policy and literature reviewed in our earlier work [33, 45], instead of originating from one of the interviews or focus group. In this project, our team performed a rapid review on virtual health care guidance published in the literature, through the lens of the Quadruple Aim Framework. The Quadruple Aim framework guided this search through its focus on four key principles when designing and delivering health care services: improving patient experience, improving population health, reducing health care costs and improving provider experience [48]. For example, Standard 1, which calls for health care providers to perform standardized symptom assessments for their patients, originated from the research found through the rapid review [33] as well as policy mandating that physicians continue to monitor their patients’ symptoms despite being based virtually [49, 50]. This type of assessment is based on the concept of measurement-based care. Measurement-based care can be described as “…routinely administered outcome measures with practitioner and patient review to inform clinical decision-making” [51]. It has been reported to be viewed favourably among Canadian clinicians delivering psychiatric care, so long as it can be implemented through methods considering ease-of-use and feasibility [52]. Although measurement-based care has been found to be effective to improve symptoms of anxiety and depression [51], it also provides a method to collect and retain a longitudinal, comprehensive record of patients’ health and symptoms in the absence of in-person tests–a useful tool to use when providing health care virtually.

However, there were also key areas in which our findings differed from other work in this area. These differences are valuable as they inform further research. For example, one report described the importance of coordinating virtual care services from local and regional perspectives to ensure that the unique circumstances and needs of different regions are taken into account [44]. Surprisingly, this did not come up during the focus group, interview or team meetings. Nevertheless, this is important to meet the needs of patients and health care providers across Canada. Another report encouraged providers to self-assess their capacity and aptitude to deliver virtual primary care services [31]. Capacity and aptitude can be built through workplace training initiatives around virtual health care. This is an important aspect of virtual health care delivery, as providers must have the tools and learning they require to successfully conduct virtual mental health appointments.

Another area for further research may explore how to ensure that episodic appointments (e.g., ‘walk-in’ services) ensure patient continuity of care. This is an important step for improving virtual care services, especially given that reports have critiqued walk-in virtual primary care services for putting patients in more danger of experiencing harm [5, 28]. For example, physicians delivering care virtually may be unable to link patients to critical community resources because the physician is not that patient’s regular healthcare practitioner and may be unaware of the patient’s background or neighbourhood [5].

### Strengths and limitations

The overarching goal of this project was the development of virtual primary mental health care standards based on the opinions of health care practitioners, people with lived experience and other community stakeholders in Canada. We created a list of standards that reflected a wide range of opinions. Consensus-driven approaches, like the one we employed, have also been used frequently across different studies discussing standard development for primary mental health care, particularly among younger populations [53–56]. A second major strength of this project was the emphasis on assessing the feasibility and agreeability of each standard with the advisory group. This allowed for standards to be debated, revised and changed to better meet the needs of patients in Canada.

Limitations included the fact that the focus group and interviews recruited a small number of participants (n = 7) and the Delphi process also only involved seven external advisory group members (there was no overlap between those two groups), which limited the range of discussions and opinions. Although the opinions and feedback expressed within this manuscript represent diversity in terms of roles within primary mental health care, respondents from non-urban environments were unrepresented. Additionally, in our original plan, we had intended to execute a formal Delphi panel using panelists that were entirely external to our team. However, due to time and resource constraints, we instead used the same anonymous Delphi process with members of the advisory group. We feel that this was an appropriate use of the process, because the members of our team were located in different regions (e.g. provinces, countries, etc.) and worked in somewhat different fields (e.g. academia, government, etc.), allowing us to maximise feedback received from this group while minimising group think. Moreover, to minimise the bias that would have come from authors reviewing standards that they wrote, none of the authors who took part in drafting the initial standards participated in any of the rounds of the Delphi consensus. Additionally, because of the afore-mentioned constraints, we were only able to implement one formal round of critiques assessing the feasibility and acceptability through the Delphi process, instead of mirroring the two rounds of critiques completed by Eubank et al. [41].

## Conclusions

The standards developed in this project can be used by primary care providers, health care organizations, and policymakers as a benchmark for what constitutes high quality virtual delivery of mental health services in Canadian primary care. Given that mental health issues are the most common reason for presentation to primary care providers, reducing unwarranted variation in care for these important issues is an important aim for health care in Canada. Future work should explore implementation of these standards into diverse primary care settings.

## Supporting information

S1 Table Consensus reporting items for studies in primary care- the CRISP statement. (DOCX)

S1 Text Focus group and interview guide. (DOCX)
