# Supplementary material for: Developing standards for virtual delivery of mental health services in Canadian primary care: A qualitative study and modified Delphi process
Source: PLOS Ment Health. 2024 Oct 17;1(5):e0000071. doi: 10.1371/journal.pmen.0000071 (PMC12798391; doi:10.1371/journal.pmen.0000071)
Supplement: S1 Table — (DOCX) [file pmen.0000071.s001.docx]

Supplementary Table 1: Consensus Reporting Items for Studies in Primary Care- the CRISP Statement

Article title: *Developing standards for virtual delivery of mental health services in Canadian primary care: a qualitative study and modified Delphi process*

| **Reporting Section** | **Item Number** | **Reporting Item Statement** | **Location Where Reporting Item is Located** |
| --- | --- | --- | --- |
| Include “primary care” and/or discipline-specific terms in the title, abstract, and/or keywords. | 1 |  | Title and abstract |
| Describe the study rationale and importance for primary care | 2a | Explain the rationale for the research question and how it relates to primary care | Abstract and background |
|  | 2b | Describe the importance or relevance of the topic under study in the primary care setting | Abstract and background |
|  | 2c | Identify any theory, model, or framework used and explain why it is appropriate to the research question in primary care | N/A – no theories, models or frameworks were used. |
| Describe the research team's primary care experience and collaboration | 3a | Describe the research team's expertise and experience in primary care practice and/or research | Middle of ‘Methods’ section |
|  | 3b | Describe whether and how primary care patients, practicing clinicians, community members, or other stakeholders were involved in the research process | Middle of ‘Methods’ section |
| Describe the study participants and populations in the context of primary care. | 4a | Use person-focused language to refer to the research populations and participants, or use terms based on patient preferences | End of ‘Methods’ section |
|  | 4b | If reporting personal characteristics of participants, report the source of the data, the rationale for using it, and the rationale for any classifications used | N/A |
|  | 4c | Describe the participants and populations in sufficient detail to allow comparison to other primary care patient populations | End of ‘Methods’ section |
|  | 4d | Specify if participants have pre-existing therapeutic relationships with the clinical team or are new patients | N/A |
| Describe the conditions under study in the context of primary care | 5a | Describe if the condition under study is acute or chronic | Mental illness can be either chronic or acute – described in Background and Results |
|  | 5b | Report how multimorbidity is considered and how it might affect the interpretation of the study findings/results | N/A |
| Describe the clinical encounter under study in the context of primary care | 6a | Specify if the study focus is an isolated clinical encounter or a longitudinal course of care. If it is an isolated clinical encounter, specify if it is the first visit or a follow-up visit for the condition under study | N/A- there were no clinical encounters with participants. |
| Describe the patient care team | 7a | If care is delivered by teams, describe the team members and their roles | N/A- no care was delivered to participants in this study. |
|  | 7b | For each clinician category, report profession, specialty, and qualifications | Start of ‘Results’ |
| Describe the study interventions in the context of primary care | 8a | Describe interventions and their implementation in sufficient detail to enable the reader to assess applicability in their own setting | N/A - no interventions were provided. |
|  | 8b | Describe any clustering or grouping of patients, participants, clinicians, teams, or practices and how it was addressed in the analysis | N/A |
|  | 8c | Describe the healthcare system in sufficient detail to allow comparisons to other systems | In ‘Background’ and in ‘Discussion’ |
| Describe study measures used and their relevance to primary care | 9a | Report if study measurement tools have been validated in primary care populations or settings | N/A - no study measurement tools were used |
|  | 9b | Describe how the measurement tools used are meaningful to primary care patients and their care | N/A |
|  | 9c | Report findings/results in forms that are clinically interpretable by primary care clinicians and patients | Findings and subsequent standards are reported in ‘Results’ and in Table 2. |
| Discuss the meaning of study findings/results in the context of primary care | 10a | Discuss implications of the study findings/results for research, patient care, education, and policy with specific focus on primary care | In ‘Discussion’ |
|  | 10b | Discuss the implications of study recommendations on demands and priorities in primary care practice | In ‘Discussion’ |
|  | 10c | Comment on any research processes that might influence the applicability of the study findings/results in diverse primary care settings | In last paragraph of ‘Discussion’ |
