# Supplementary material for: Developing standards for virtual delivery of mental health services in Canadian primary care: A qualitative study and modified Delphi process
Source: PLOS Ment Health. 2024 Oct 17;1(5):e0000071. doi: 10.1371/journal.pmen.0000071 (PMC12798391; doi:10.1371/journal.pmen.0000071)
Supplement: S1 Text — (DOCX) [file pmen.0000071.s002.docx]

Supplemental Information 1:

Focus Group and Interview Guide

***Read out first:*** Thanks for sharing your time with us today. We’re going to start by talking about some research findings we have on this topic already, and then we will discuss your thoughts and experiences.

The first thing we did in this project is that we reviewed studies from all over the world to determine what recommendations regarding mental health care, or virtual health care alone, have already been published. After screening over 2,000 articles to check which matched our criteria, we found 40 that had information about this . We reviewed these articles to find out the information they had related to the ‘Quadruple Aims’- a framework describing four that should be considered when trying to develop any type of guideline for health care services. The key things we found that seem to be really important to support good virtual mental health care are: screen patients for appropriateness of virtual care; obtain emergency contact details; communicate transparently with patients; improve marginalised patients’ access to care; support health equity for all patients; determine the cost-effectiveness of virtual care; inform patients of insurance coverage for virtual care services; increase provider training for virtual care and set professional boundaries between providers and patients. A key takeaway from this research is the need for high quality guidelines for virtual mental health care, which can be used to guide development of training for providers. We hope that this research will be used to develop proper guidelines on how and when to deliver virtual care for mental health conditions in primary care settings across Canada.

**Introductions (*For Focus Group only*)**

Now we will start by taking a few minutes to introduce ourselves. Could you please share your name, where you are right now, and what got you interested in this project; and try to keep that to about 30 seconds each. Thank you.

**General questions**

-Is anyone willing to share some experiences with virtual mental health care? (*Alternative for interviews: “Could you start by sharing some of your experiences with virtual mental health care?”*)

-What are some things that went well?

-What are some things that have not gone very well?

-Have you noticed any differences between how mental health care is done on phone or video, between primary care providers and other health professionals?

**Specific questions (“Now we’d like to ask some more specific questions”)**

| **Questions for healthcare professionals** | **Questions for people with lived experience** |
| --- | --- |
| Have you mostly used a phone to conduct visits? Have you used video? Was one better than the other? | Have you mostly used a phone to conduct visits? Have you used video? Was one better than the other? |
| How does the consent process work before starting an appointment or some kind of virtual interaction? Are there options to choose virtual vs. in person care? How is that decided? | How does the consent process work before starting an appointment or some kind of virtual interaction? Are there options to choose virtual vs. in person care? How is that decided? |
| Do you feel that clinicians are getting adequate education about how to conduct visits and provide mental health care virtually? | Do you feel that clinicians are getting adequate education about how to conduct visits and provide mental health care virtually? |
| When resources like groups or other services are provided, how easy is it to communicate information about that? Are there challenges around figuring out what the most appropriate local resources are? | When resources like groups or other services are provided, how easy is it to communicate information about that? Are there challenges around figuring out what the most appropriate local resources are? |
| Are there things you routinely do in advance of conducting virtual mental health appointments to make sure ? During appointments? After appointments? To make sure it goes well | Are there things you routinely do in advance of your own virtual mental health appointments to make sure ? During appointments? After appointments? To make sure it goes well |
| In the setting in which you work, what if anything is done to understand patients’ specific needs (for example, different needs based on cultural background). Are patients given an opportunity to choose different providers based on some characteristic of that provider? | Do you think your mental health care provider understands your specific needs (for example, different needs based on your cultural background?) Are you given an opportunity to choose different providers based on what is most comfortable for you? |
| Do you have consistent access to the technology you need to conduct appointments? Do you have a safe, private space for appointments? | Do you have consistent access to the technology you need for appointments? Do you have a safe, private space for appointments at a time that works for you? |
| What barriers do you face in delivering virtual mental health? and what do you think can be done differently? | What barriers do you face in accessing virtual mental health? and what do you think can be done? |
| How does the consent process work? Are there options to choose virtual vs. in person care? | How does the consent process work? Are there options to choose virtual vs. in person care? |
| Do you feel there are differences between in person and virtual with respect to data security? How has this been dealt with in the settings you work in or access care in? | Do you feel there are differences between in person and virtual with respect to data security? How has this been dealt with in the settings you access care in? |

**Concluding questions (“Now we are going to ask some final questions”)**

-How could primary care providers improve virtual mental health care? What resources would they need (and from whom) to be successful?

-Is there anything else that anyone wanted to share about this topic?

Thank you
